# Multi-site observational maternal and infant COVID-19 vaccine study (MOMI-vax): a study protocol

**DOI:** 10.1186/s12884-022-04500-w

**Published:** 2022-05-12

**Authors:** Flor M. Munoz, Richard H. Beigi, Christine M. Posavad, Barbra A. Richardson, Helen Y. Chu, Karin Bok, James Campbell, Cristina Cardemil, Emily DeFranco, Robert W. Frenck, Mamodikoe Makhene, Jeanna M. Piper, Jeanne Sheffield, Ashley Miller, Kathleen M. Neuzil

**Affiliations:** 1 Departments of Pediatrics and Molecular Virology and Microbiology, Section of Infectious Diseases, Baylor College of Medicine, 1102 Bates St. Suite 1150, Houston, TX 77030 USA; 2 Department of Obstetrics, Gynecology & Reproductive Sciences, University of Pittsburgh School of Medicine, UPMC Magee-Womens Hospital, 300 Halket Street, Pittsburgh, PA 15213 USA; 3 Vaccine and Infectious Disease Division, Fred Hutchinson Cancer Research Center, and Department of Laboratory Medicine and Pathology, University of Washington, 1100 Fairview Ave N, Seattle, WA 98109 USA; 4 Department of Biostatistics, University of Washington, and Vaccine and Infectious Disease Division, Fred Hutchinson Cancer Research Center, 1100 Fairview Ave N, Seattle, WA 98109 USA; 5 Department of Epidemiology, University of Washington School of Public Health, 750 Republican St, Seattle, WA 98109 USA; 6 Office of Director, Vaccine Research Center, National Institute of Allergy and Infectious Diseases, National Institutes of Health, 40 Convent Drive, Bethesda, MD 20892 USA; 7 Center for Vaccine Development and Global Health, University of Maryland School of Medicine, 685 W. Baltimore St, Baltimore, MD 21201 USA; 8 Division of Microbiology and Infectious Diseases, National Institute of Allergy and Infectious Diseases, National Institutes of Health, 5601 Fishers Lane, Rockville, MD 20892 USA; 9 Department of Obstetrics and Gynecology, University of Cincinnati College of Medicine, 231 Albert Sabin Way, Cincinnati, OH 45267-0526 USA; 10 Department of Pediatrics, University of Cincinnati Children’s Hospital Medical Center, 3333 Burnet Ave, MLC 6014, Cincinnati, OH 45229 USA; 11 Division of AIDS, National Institute of Allergy and Infectious Diseases, National Institutes of Health, 5601 Fishers Lane, Rm 8B68, MSC 9831, Rockville, MD 20892 USA; 12 Department of Gynecology and Obstetrics, Johns Hopkins University, 600 N Wolfe St., Nelson Building 2nd floor, Baltimore, MD 21287 USA; 13 FHI 360, 359 Blackwell Street, Suite 200, Durham, NC 27701 USA

**Keywords:** Pregnant women, Postpartum women, COVID-19 vaccines, Immunogenicity, Infant immune responses

## Abstract

**Background:**

Pregnant women were excluded from investigational trials of COVID-19 vaccines. Limited data are available to inform pregnant and postpartum women on their decisions to receive a COVID-19 vaccine.

**Methods:**

The goal of this observational, prospective cohort study is to evaluate the immunogenicity and safety of various Emergency Use Authorization (EUA) or licensed COVID-19 vaccines administered to pregnant or lactating women and describe the transplacental antibody transfer and kinetics of antibodies in mothers and infants. The study is adaptive, allowing additional groups to be added as new vaccines or vaccine regimens are authorized. Up to 20 clinical research institutions in the United States (U.S.) will be included. Approximately 200 pregnant women and 65 postpartum women will be enrolled per EUA or licensed COVID-19 vaccine formulation in the U.S. This study will include pregnant and postpartum women of all ages with and without chronic medical conditions. Their infants will be enrolled and followed beginning at birth in the pregnant cohort and beginning at the earliest possible time point in the postpartum cohort. Blood samples will be collected for immunogenicity outcomes and pregnancy and birth outcomes assessed among women and infants. Primary analyses will be descriptive and done by vaccine type and/or platform.

**Discussion:**

Given the long-standing and legitimate challenges of enrolling pregnant individuals into clinical trials early in the vaccine development pipeline, this study protocol describes our current study and provides a template to inform the collection of data for pregnant individuals receiving COVID-19 or other vaccines.

**Trial registration:**

NCT05031468.

**Supplementary Information:**

The online version contains supplementary material available at 10.1186/s12884-022-04500-w.

## Background

Pregnant women are susceptible to infection and complications from SARS-CoV-2. Observational data in the U.S. demonstrate that, while the absolute risk for severe health effects may be low, pregnant women have an increased risk of developing severe COVID-19, including illness that results in pneumonia, intensive care unit (ICU) admission, need for mechanical ventilation, and death compared with non-pregnant women of reproductive age with COVID-19 [1–5]. Pregnant women who acquire COVID-19 are more likely to experience adverse consequences including preterm birth, and their infants have an increased risk of neonatal intensive care unit (NICU) admission [2, 4, 6–9]. Maternal immunization may be a strategy to provide protection to both the mother and the infant against complications associated with COVID-19.

Several COVID-19 vaccines utilizing different platforms (e.g., mRNA, viral vectored, protein-based, inactivated virus), are available or will soon be available under EUA in the U.S. or will soon be licensed. Pregnant women were not included in pivotal efficacy trials of these vaccines, and currently, only the Pfizer/BioNTech mRNA vaccine is being tested in a randomized controlled trial among pregnant women [10]. Conducting placebo-controlled trials in pregnant women is increasingly problematic given the widespread availability of these vaccines in the U.S. and many other countries, where approved vaccines are being administered to individuals who are pregnant or postpartum, as part of national vaccination campaigns. Safety data are being collected through various surveillance systems in the U.S., with reassuring results to date [11, 12]. However, many knowledge gaps remain regarding the use of COVID-19 vaccines during pregnancy and after delivery. Data on kinetics and durability of maternal antibodies, transplacental antibody transfer to the infant, breast milk antibodies, and kinetics of antibodies in infants, are limited [13, 14].

We designed an observational study in pregnant and postpartum women in order to fill knowledge gaps on use of COVID-19 vaccines in these populations. The purpose of this manuscript is to share our rationale and study design, laboratory evaluations, and analyses plans, and to serve as a template for similar studies of COVID-19 and other vaccines in pregnant and postpartum populations (including those who are lactating). It is expected that the results of this study will inform policy recommendations and personal decision-making on the use of approved COVID-19 vaccines in pregnant and postpartum individuals in the U.S. and globally.

### Trial overview

The goal of this observational, prospective cohort study is to evaluate the immunogenicity and safety of various EUA or licensed COVID-19 vaccines administered to pregnant or lactating women and describe the transplacental antibody transfer and kinetics of antibodies in mothers and infants. The adaptive study design allows the evaluation of up to 5 vaccines, with groups added as new vaccines are authorized and vaccination recommendations evolve.

## Methods

Approximately 1000 total study participants who receive or plan to receive a COVID-19 vaccine during pregnancy and/or postpartum and their infants will be enrolled. Pregnant women during any trimester of pregnancy will be enrolled either prior to or after receipt of vaccine(s) and followed with periodic blood draws for immunogenicity assessments. Pregnancy status will be confirmed through medical records, and vaccination status will be confirmed through medical records or vaccination card. Infants will be enrolled at birth. At that time, maternal and cord blood samples will be obtained, and details of the birth will be collected. Mother and infants will be followed through 12 months with periodic immunogenicity sampling and safety follow-up. Thus, the study duration of participation for pregnant women will be up to 20 months. A cohort of postpartum women will be enrolled either prior to or after receipt of vaccine(s) within the first 2 months of delivery. Their infants will be enrolled at the same time and will be followed through 12 months with periodic immunogenicity sampling and safety follow-up. A schedule of events is found in Tables 1 and 2.

**Table 1:**
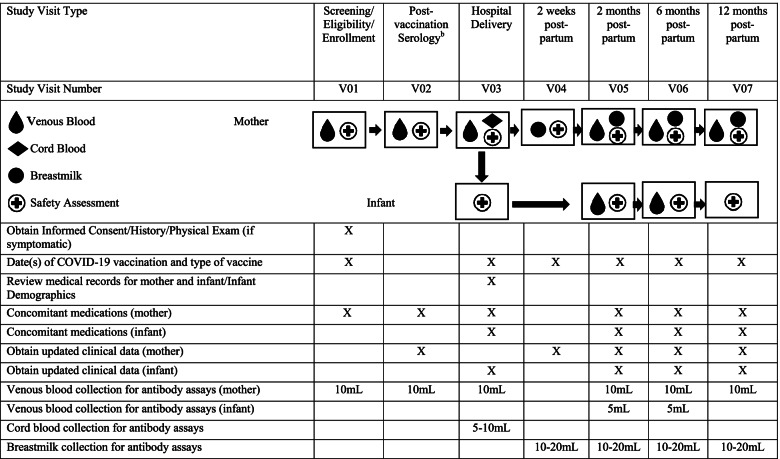
Schedule of Events for Multi-site Observational Maternal and Infant COVID-19 Vaccine Study (MOMI-Vax). Schedule of Events: Pregnant Women and their Infants^a^

*Abbreviations*: *mL* milliliters

^a^*n* = approximately 200 pregnant women who are scheduled to receive or have completed any licensed or EUA COVID-19 vaccine series per vaccine type and approximately 200 infants born to pregnant women per vaccine type

^b^Only applicable if V01 was completed pre-vaccination

**Table 2:**
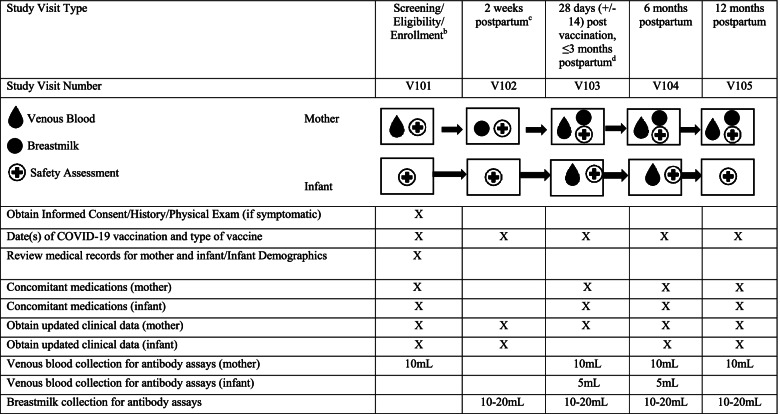
Schedule of Events for Multi-site Observational Maternal and Infant COVID-19 Vaccine Study (MOMI-Vax). Schedule of Events: Postpartum Women and their Infants^a^

*Abrreviations*: *mL* milliliters

^a^*n* = approximately 65 postpartum women who are scheduled to receive or have initiated any licensed or EUA COVID-19 vaccine series within first 2 months of delivery; *n* = approximately 65 infants of postpartum women

^b^For mothers vaccinated prior to enrollment: Document COVID-19 vaccination date(s) and type

^c^Optional, for mothers enrolled at delivery

^d^If visit overlaps with Visit 101 (i.e., mother received full series prior to enrollment), the two visits will be combined

Written informed consent will be obtained from the pregnant and postpartum participants at the enrollment visit. Informed consent will be obtained from the mother for the infant either at the time of birth or for postpartum individuals, at time of enrollment (see Additional Files 1, 2 and 3). This study will be carried out in accordance with Good Clinical Practice (GCP) guidelines. The Vanderbilt University Medicine Center Institutional Review Board (IRB) has reviewed and approved this protocol, consent forms and associated documents. Each institution engaged in this research will hold an Office of Human Research Protections (OHRP)-approved Federal Wide Assurance (FWA). All study records will be kept in a locked file cabinet or maintained in a locked room at the site. Electronic files will be password-protected. Only people who are involved in the conduct, oversight, monitoring, or auditing of this study will be allowed access to the PHI that is collected. Any publications from this study will not use information that will identify subjects by name. Organizations that may inspect and/or copy research records maintained at the site for quality assurance (QA) and data analysis include groups such as the local or central IRB, NIAID, and the FDA.

Quality oversight procedures will be implemented by FHI 360 in consultation with the Infectious Disease Clinical Research Consortium (IDCRC) Clinical Operations Unit (COU). Quality oversight will include review of each participating site’s Clinical Quality Management Plan - Quality Management Summary Report on a quarterly basis, as well as overall enrollment tracking and oversight of performance trends.

### Study population

This observational study was designed to be broadly inclusive to reflect the need to collect information on pregnant and postpartum women of all ages, with and without chronic medical conditions, and with singleton or multiple gestations. The study will include postpartum women whether or not they choose to breastfeed. The exclusion criteria are limited to behavioral or cognitive impairment, psychiatric disease or any condition that might pose a health risk and interfere with the individual’s ability to participate and/or the evaluation of study objectives (Table 3).

**Table 3:** Study Inclusion and Exclusion Criteria

**Inclusion Criteria**	Pregnant Women 1. Pregnant individuals scheduled to receive or have received complete vaccination series of any licensed or EUA COVID-19 vaccine^a^ 2. Willing and able to provide consent for study participation for herself and for her infant prior to initiation of any study procedures.
	Postpartum Women 3. Individuals scheduled to receive or who have initiated vaccination series of any licensed or EUA COVID-19 vaccine within the first 2 months postpartum^b^ 4. Willing and able to provide consent for study participation for herself and for her infant prior to initiation of any study procedures (a separate consent form will be used for their infants).
	All participants 5. Understands and agrees to comply with all study procedures. 6. Agrees to sign medical release for herself and her infant to allow study staff to gather pertinent medical information, pregnancy outcome data, and medical information as needed.
**Exclusion Criteria**	1. Behavioral^c^ or cognitive impairment or psychiatric disease that, in the opinion of the investigator, may interfere with the participant’s ability to participate in the study. 2. Any condition which, in the opinion of the investigators, may pose a health risk to the participant or interferes with the evaluation of the study objectives

*Abbreviations*: *EUA* Emergency Use Authorization

^a^No limitation on age of mother, health status, or gestational age at enrollment

^b^No limitation on maternal age or health status

^c^Includes having a history of alcohol or drug abuse within 1 year prior to study enrollment

## Study procedures

### Recruitment and screening

Participants will be recruited from up to 20 academic affiliated, IDCRC sites throughout the U.S. Study staff will inform potential participants/legally authorized representatives of the study, obtain informed consent, and determine study eligibility.

### Enrollment

Screening and enrollment visits will occur sequentially or on the same day. Study staff will collect baseline information on participants (e.g., demographics, medical history, obstetric history, history of respiratory illnesses) (Tables 1 and 2). Pregnant individuals who meet all eligibility criteria will be enrolled. Infants born to pregnant individuals will become study participants upon delivery and maternal consent. Postpartum individuals who meet all eligibility criteria will be enrolled along with their infants. Once enrolled, each individual will be registered in a study database. Enrollment will continue until number of participants is reached for a particular vaccine type/study group. If new vaccines receive EUA or licensure, vaccine types will be added.

### Follow-up

For women enrolled during pregnancy, a pre- and post-vaccination serology will be obtained if the subject had not received a vaccine prior to screening/enrollment, and post-vaccination serology will be obtained at any time after receipt of vaccine at the time of screening/enrollment if the subject had previously completed their full vaccination series. Routine follow-up visits for the women and infants include hospital delivery date, 2 weeks, 2, 6, and 12 months postpartum. Routine visits for postpartum women and their infants include visits at 2 weeks postpartum (or earliest enrolled date), 28 days (+/− 14) after last vaccination/ ≤3 months postpartum, 6 and 12 months postpartum. All routine visits will consist of obtaining updated clinical data, concomitant medications, collection of venous blood for antibody assays, obtaining date(s)/type of COVID-19 vaccine, date and severity of confirmed COVID-19 illness, and optional collection of breast milk samples (Tables 1 and 2). Unscheduled visits may be needed to complete study procedures that could not be conducted at the routine visits.

### Study outcomes

Outcomes of pregnancy, both maternal and fetal/infant, will be collected. Since this is an observational protocol, no serious adverse events (SAEs) or adverse events of special interest (AESIs) will be collected during this study. Potential non-serious adverse events that could occur in association with study procedures such as blood sample collection will be reported to the IRB. Maternal outcomes that will be recorded include death of mother or fetus, maternal hospitalization or prolonging of existing hospitalization, or important medical events reported after vaccination requiring treatment. Infant outcomes that will be recorded include neonatal or infant death through 12 months post-delivery, infant hospitalization, a congenital defect or genetic anomaly, preterm delivery/prematurity (defined as live birth prior to 37 weeks gestation). The assessment of these maternal and infant events will be harmonized by using existing Brighton Collaboration case definitions [15].

Pregnancy outcome data include the type of delivery (e.g., vaginal vs. Cesarean section), and any complications during labor and delivery for both the mother as well as the neonate. Neonatal assessments include but are not limited to gestational age, birth weight, Apgar scores, congenital and genetic abnormalities, infection, hematological and metabolic complications, admission to nursery or NICU and the need for respiratory support or other life sustaining interventions.

All information on COVID-19 cases during the study will be collected via medical records including polymerase chain reaction (PCR) or antigen testing results and severity of illness.

### Laboratory assays

#### Serum

Primary and secondary endpoint assays to determine serum levels of total and SARS-CoV-2 antigen-specific immunoglobulin G (IgG) [16] and live [17] and pseudovirus [ 18] neutralizing antibodies will be performed at central IDCRC laboratories utilizing qualified assays (Table 4). Reagents will include vaccine-matched and emerging variants to evaluate the ability of variants to escape antibody generated by vaccine in this cohort. Additional exploratory endpoint analyses may include Ig subclass, and SARS-CoV-2 IgG Fc glycosylation (Table 4) [20].

**Table 4:** Assays

Study Endpoint	Specimen	Assay	Readout
Primary	Venous and Cord Blood Serum	MSD**®** V-Plex SARS-CoV-2 Panel 2 [16]	Spike, RBD and N IgG binding Ab
		FRNT-mNG [17]	Ab neutralizing SARS-CoV-2
		Pseudoneutralization [18]	Ab neutralizing SARS-CoV-2
Secondary	Breast Milk	MSD**®** V-Plex SARS-CoV-2 Panel 2 [16]	Spike, RBD and N IgG and IgA binding Ab
		FRNT-mNG [17]	Ab neutralizing SARS-CoV-2
		Pseudoneutralization [18]	Ab neutralizing SARS-CoV-2
Exploratory	Venous and Cord Blood Serum; Breast Milk	MSD**®** V-Plex SARS-CoV-2 Panel 2 [16] – emerging variant reagents	Vaccine-induced Ab binding to emerging variants
		FRNT-mNG [17] - emerging variant reagents	Vaccine-induced Ab neutralizing emerging variants
		Pseudoneutralization [18] - emerging variant reagents	Vaccine-induced Ab neutralizing emerging variants
		Luminex [19] or MSD**®**	Antigen-specific Ab isotype and subclass profiles
		IgG glycan analysis [20]	IgG Fc glycosylation
Ancillary^a^	Whole Blood	RNAseq, single-cell RNAseq [21]	Gene signatures to identify vaccine biomarkers
	PBMC	ELISpot/flow cytometry [22–24]	T and B memory cells, plasmablasts
		ICS/flow cytometry [25]	CD4/CD8 T cells
		Tetramer-staining	SARS-CoV-2 specific T cells
		MSD**®** cytokine panel	Th1/Th2 cytokines

*Abbreviations*: *MSD* Meso Scale Discovery®, *RBD* receptor binding domain, *FRNT-mNG* Focus Reduction Neutralization Test mNeonGreen, *Ab* antibody, *ICS* intracellular cytokine staining

^a^Collection of whole blood and PBMC under consideration in a subset of participants and/or at a subset of sites

#### Breast milk

Measurement of total and SARS-CoV-2 antigen-specific immunoglobulin IgA (IgA) and IgG and live and pseudovirus neutralizing antibodies will be performed at the same central laboratories as with serum (see above and Table 4). These assays are being qualified using breast milk samples from vaccinated and/or COVID-19 infected women.

#### Other samples

The collection of whole blood and its processing for peripheral blood mononuclear cells (PBMC) in a subset of participants and/or subset of clinical sites are under discussion in order to conduct additional ancillary studies (Table 4).

### Data collection, quality and management

Data collection is the responsibility of the study personnel at the participating clinical study site under the supervision of the site PI. During the study, the site PI must maintain complete and accurate documentation for the study.

The site PI is responsible to ensure the accuracy, completeness, legibility, and timeliness of the data reported. All source documents should be completed in a neat, legible manner to ensure accurate interpretation of data.

Copies of the electronic Case Report Form (eCRF) will be provided for use as source CRFs, as needed, and maintained for recording data for each subject enrolled in the study. Data reported in the eCRF derived from source CRFs should be consistent or the discrepancies should be explained.

The data coordinating center for this study will be responsible for data management, quality review, analysis, and reporting of the study data.

Clinical (including, but not limited to, AEs, concomitant medications, medical history, physical assessments) and immunogenicity data will be entered into a 21 CFR 11-compliant Clinical Data Management System provided by the data coordinating center. The data system includes password protection and internal quality checks, such as automatic range checks, to identify data that appear inconsistent, incomplete, or inaccurate. Clinical data will be entered directly from the CRFs completed by the study personnel.

### Statistics

This study will generate descriptive data that is supportive of the hypothesis that COVID-19 vaccines elicit adequate immune responses among pregnant and postpartum individuals and will describe the transplacental antibody transfer for SARS-CoV-2 and serum SARS-CoV-2 antibodies in mother and infants of vaccinated mothers.

**Fig. 1:**
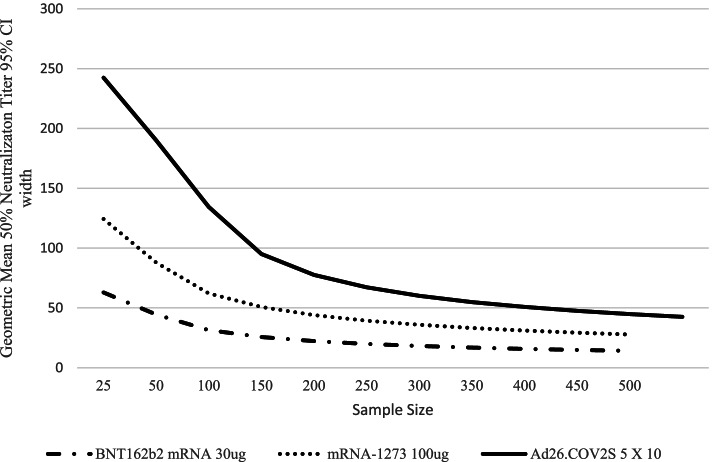
Sample Size Calculation based on Width of 95% Confidence Intervals for Neutralizing Antibodies for COVID-19 Vaccines included in MOMI-Vax. Legend: The curve shows neutralizing antibody from Phase 1 studies and demonstrates the inflection point for sample size at approximately 150 individuals per group, with little gain in precision with higher samples sizes. Results similar using binding antibody data (results not shown)

Approximately 200 pregnant individuals and their infants and up to 65 postpartum individuals and their infants per SARS-CoV-2 vaccine type (Groups 1–4) will be enrolled in this protocol. Two additional groups (Groups 5 and 6) have been added since beginning enrollment following approval of administration of a booster dose in late 2021. It is important to generate data on kinetics and durability of maternal and infant antibodies for all vaccine regimens, including those with additional doses beyond the primary series administered during pregnancy. Figure 1 illustrates the study precision of estimation of GMT of Neut antibodies the study with various sample sizes using Phase 1 data from the Pfizer, Moderna, and Janssen COVID-19 vaccines [26–28]. In addition, there will be good precision for estimation of transplacental transfer ratios.

The analysis groups are:

- Group 1: Individuals who receive a COVID-19 vaccine during pregnancy (up to 200 individuals per vaccine type).
- Group 2: Individuals who receive a COVID-19 vaccine postpartum (up to 65 individuals per vaccine type).
- Group 3: Infants of individuals who receive COVID-19 vaccine during pregnancy (approximately 200 infants per vaccine type).
- Group 4: Infants of individuals who receive COVID-19 vaccine postpartum (approximately 65 infants per vaccine type).
- Group 5: Individuals who receive additional COVID-19 vaccine(s) (booster) during pregnancy (up to 200 individuals).
- Group 6: Infants of individuals who received additional COVID-19 vaccine(s) (booster) during pregnancy (approximately 200 infants).

The study will not randomize participants between vaccines, and statistical analyses will seek to adjust for covariates when deemed appropriate to account for potential confounders. All analyses will be done by vaccine type and/or platform or vaccination regimen on all available data, and analyses for each endpoint are outlined in Table 5. Multiple imputation may be used if there is substantial missingness. Additional analysis details will be included in a statistical analysis plan.

**Table 5:** Primary, Secondary and Exploratory Objectives, Endpoints and Analyses

	Objectives	Endpoints	Analyses
**Primary**	1. Immunogenicity: To describe kinetics and durability of maternal serum antibodies following receipt of COVID-19 vaccine in individuals vaccinated during pregnancy, by vaccine type and platform.	• Geometric Mean Titer (GMT) of serum immunoglobulin G (IgG) enzyme-linked immunosorbent assay (ELISA) and neutralizing (Neut) antibodies after vaccination and up to 12 months after delivery, by vaccine type and platform^a^	• Geometric Mean Titers (GMTs) and 95% Confidence Intervals (CIs) will be calculated in Group 1. Plots such as reverse cumulative distributions or longitudinal presentations of GMTs will be presented.
	2. Immunogenicity: To describe the transplacental antibody transfer of SARS-CoV-2 antibodies among individuals vaccinated during pregnancy, overall and by vaccine type and platform.	• GMT and ratio of cord blood to maternal serum IgG ELISA and Neut antibodies overall and by vaccine type and platform.	• GMTs and ratio of cord blood to maternal serum and 95% CIs will be calculated in Group 1.
	3. Immunogenicity: To describe kinetics and durability of serum SARS-CoV-2 antibodies in infants of mothers vaccinated during pregnancy, by vaccine type and platform.	• GMT of serum IgG ELISA and Neut antibodies at birth (cord blood) and at approximately 2 and 6 months of age in all infants, by vaccine type and platform.	• GMTs and 95% CIs will be calculated in Group 3. Plots such as reverse cumulative distributions or longitudinal presentations of GMTs will be presented.
**Secondary**	1. Safety: To describe pregnancy outcomes in individuals who receive COVID-19 vaccine during pregnancy or postpartum, and their infants, by vaccine type and platform.	• Frequency of maternal and infant outcomes vs. background rates in the US, overall and by vaccine type and platform.	• A comparison of frequencies to background rates will be reported in Groups 1–4 (no formal statistical testing).
	2. Immunogenicity: To evaluate immune responses and durability of antibodies in individuals who received different COVID-19 vaccines during pregnancy or postpartum compared to non-pregnant populations of women of childbearing age and to a correlate of protection (if available), by vaccine type and platform.	• GMT of serum IgG ELISA and Neut antibodies after vaccination in pregnancy or postpartum vs. mean titers achieved by non-pregnant participants in clinical trials and to a correlate of protection if available, by vaccine type and platform.	• One sample t-tests will be done in Group 1.
	3. Immunogenicity: To assess the effect of gestational age at vaccination (trimester of gestation), maternal age, health and risk status, on immune responses and durability of antibodies in individuals who receive different COVID-19 vaccines during pregnancy or post-partum, by vaccine type and platform.	• GMT of serum IgG ELISA and Neut antibodies after vaccination in pregnancy by gestational age at vaccination (trimester of gestation) and interval between vaccination and delivery, by vaccine type and platform. GMT of serum IgG ELISA and Neut antibodies after vaccination in pregnancy or postpartum by maternal age, health status, and risk status (e.g., occupation, priority vaccination group) overall and by vaccine type and platform.	• GMTs and 95% CIs will be calculated for Group 1 by strata. Student’s t-tests and linear regression (controlling for potential confounders) will be used to compare strata.
	4. Immunogenicity: To describe the transplacental antibody transfer of SARS-CoV-2 antibodies among individuals vaccinated during pregnancy, by gestational age at vaccination, by interval from vaccination to delivery and maternal age, health and risk status, overall and by vaccine type and platform.	• GMT and ratio of cord blood to maternal serum IgG ELISA and Neut antibodies at delivery by gestational age at vaccination (trimester of gestation) and interval between vaccination and delivery, overall and by vaccine type and platform. • GMT and ratio of cord blood to maternal serum IgG ELISA and Neut antibodies at delivery by maternal age, health status, and risk status, overall and by vaccine type and platform.	• GMTs and ratios of cord blood to maternal serum and 95% CIs will be presented for Group 1 by strata. Student’s t-tests and linear regression (controlling for potential confounders) will be used to compare strata.
	5. Immunogenicity: To describe the kinetics of SARS-CoV-2 antibodies in breast milk of mothers who received vaccine during pregnancy or postpartum, overall and by vaccine type and platform.	• GMT of IgG and immunoglobulin A (IgA) ELISA and Neut antibodies in breast milk at approximately 2 weeks, and 2, 6, and 12 months postpartum, in individuals vaccinated during pregnancy or postpartum, overall and by vaccine type and platform.	• GMTs and 95% CIs will be calculated in Group 1. Plots such as reverse cumulative distributions or longitudinal presentations of GMTs will be presented.
	6. Immunogenicity: To describe the kinetics and durability of maternal serum antibodies following receipt of COVID-19 vaccine in individuals vaccinated postpartum, by vaccine type and platform.	• Postpartum individuals: GMT of serum IgG ELISA and Neut SARS-CoV-2 antibodies pre- and/or post-vaccination, and at approximately 2, 6, and 12 months after delivery, overall, and by vaccine type and platform.	• GMTs and 95% CIs will be calculated in Group 3. Plots such as reverse cumulative distributions or longitudinal presentations of GMTs will be presented.
	7. Immunogenicity: To describe the kinetics and durability of serum SARS-CoV-2 antibodies in infants of individuals vaccinated postpartum, by vaccine type and platform.	• GMT of serum IgG ELISA and Neut antibodies at approximately 2 and 6 months of age in infants of postpartum individuals, overall and by vaccine type and platform.	• GMTs and 95% CIs will be calculated in Group 4. Plots such as reverse cumulative distributions or longitudinal presentations of GMTs will be presented.
**Exploratory**	1. To describe the effectiveness of COVID-19 vaccines against maternal COVID-19 infection during pregnancy and postpartum.	• Incidence of laboratory confirmed COVID-19 and severity of disease during study participation assessed through passive surveillance in individuals vaccinated during pregnancy or postpartum vs. rates in unvaccinated population of women of childbearing age, overall and by vaccine type and platform.	• Incidence rates and 95% CIs will be calculated in Groups 1 and 3.
	2. To describe the effectiveness of maternal antibodies to provide protection against SARS-CoV-2 infection/symptomatic disease/severity in infants in the first 12 months of life.	• Incidence of laboratory confirmed COVID-19 and severity of disease during study participation assessed through passive surveillance in infants of individuals vaccinated in pregnancy or postpartum vs. background rates in infants of unvaccinated population, overall and by vaccine type and platform.	• Incidence rates and 95% CIs will be calculated in Groups 2 and 4.
	3. To describe the effectiveness of breast milk antibodies to provide protection against SARS-CoV-2 infection/symptomatic disease/severity in infants in the first 12 months of life.	• Incidence of laboratory confirmed COVID-19 and severity of disease during study participation through passive surveillance in breastfed infants vs. not breastfed infants, by vaccine type and platform. • Incidence of laboratory confirmed COVID-19 and severity of disease during study participation through passive surveillance in breastfed infants of individuals vaccinated during pregnancy vs. postpartum, overall and by vaccine type and platform.	• Incidence rates and 95% CIs will be calculated in Groups 2 and 4.

*Abbreviations*: *GMT* Geometric Mean Titer, *IgG* immunoglobulin G, *ELISA* enzyme-linked immunosorbent assay, *Neut* neutralizing, *IgA* immunoglobulin A

^a^Pregnant individuals: pre- and/or post-vaccination, at delivery, and at approximately 2, 6, and 12 months postpartum

## Discussion

The observational and opportunistic study design outlined in this paper will provide meaningful data within the context of the current state of the ongoing pandemic due to SARS CoV-2 in pregnant women and newborns. The favorable decision by the Centers for Disease Control and Prevention (CDC), the Food and Drug Administration (FDA), and the American College of Obstetricians and Gynecologists (ACOG) in late 2020 to adopt a permissive approach, paving the way for pregnant and breastfeeding individuals to make a personal choice to take the available mRNA vaccines, was a major step forward in the inclusion of pregnant women in vaccine implementation early in the pandemic [3, 29–32]. However, it did present the frequent and suboptimal situation of women weighing the decision to choose the intervention based on an overall paucity of data. Thus, pregnant individuals are continuing to weigh the decision to receive these vaccines under EUA in a setting of limited clinical trial data on safety and efficacy specific to this population despite the reassuring observational data that are accumulating [11, 33].

At this point in the pandemic multiple vaccines are available via EUA, and thousands of pregnant and lactating individuals are opting to take them. This fact makes prospective placebo-controlled trials in pregnant and lactating persons challenging to conduct at the present time. Many within the fields of maternal/neonatal health, reproductive infectious diseases, and biomedical ethics believe the ideal approach for COVID-19 (and future emerging infectious diseases) could have included contemporaneous enrollment of pregnant and lactating individuals into clinical sub-studies in the late summer and fall of 2020 of appropriate vaccine candidates that had cleared the mandatory animal studies known as basic Developmental and Reproductive Toxicology (DART) studies as well as early human Phase 1 and II studies [34]. It is appreciated that such an approach would have necessitated special consideration and attention early in the process of vaccine development and clinical trial planning. However, it would have undoubtedly provided much needed data to optimize provider counseling and maternal decision making for this population at high risk for adverse outcomes from COVID-19 [1, 2]. There are no compelling reasons to believe the mRNA-based non-live vaccines pose real harm to women or their unborn babies and the emerging observational registry data from the CDC also demonstrate a highly favorable risk-benefit ratio of these vaccines [9]. However, uptake (and thus disease prevention) in this high-risk population has been suboptimal, due in part to the lack of information and the ambiguity around safety in pregnancy. In absence of clear data (and thus clear recommendations) care providers are left to fill in data gaps with anecdotal and/or personal beliefs that may complicate decision making for pregnant persons. While that approach can sometimes be helpful for individual patients, on the whole it is suboptimal and leads to haphazard public health practices. We believe the current protocol format offers an appropriate approach to build on what can be currently done to inform this important data gap for healthcare providers, pregnant individuals, and their babies.

This protocol template has additional advantages for prospective consideration for the current and/or future emerging infectious diseases outbreaks. Given the long-standing and legitimate challenges of enrolling pregnant individuals into clinical trials early in the drug and vaccine development pipeline, this adaptive design can help gather useful data for pregnant individuals that are being offered interventions. These populations often suffer from a disproportionate impact from infectious diseases and having the ability to rapidly design and roll-out an opportunistic approach allows for more rapid data gathering that can inform patient and provider decision making as the epidemic unfolds. This design also serves as a template for investigating current and future global vaccine dissemination, immunogenicity, and safety in pregnancy. Such an approach becomes especially relevant with the associated global variability in vaccine availability and logistical realities affecting supply and distribution, with attention to low and middle-income countries. The protocol outlined herein is adaptable to whichever vaccine products are available in varied locations, making it an attractive way to garner global data that can feed-forward and drive prospective vaccine uptake with demonstrations of immunogenicity and safety for various vaccine platforms. For example, when booster doses of COVID-19 vaccines were recommended in the US, we amended this protocol to include a group of pregnant women receiving booster doses of vaccine. Lastly, use of the Brighton Collaboration standardized case definitions for studies conducted in pregnancy further adds to the value of this work as a template for future investigations [15].

The field of research in pregnant and lactating individuals has many real and perceived challenges. It is our collective belief and hope that this protocol design can better inform and thus drive optimal decision making for all stakeholders during the ongoing COVID-19 global pandemic and future outbreaks as well. Further, the adaptive design allows for additional of new vaccines as well as evaluation of booster doses or combination vaccines as recommendations evolve.

## Trial status

This paper reflects Protocol Version 1.0, April 12, 2021 and relevant amendment for the addition of a pregnancy booster group. Recruitment began on July 6, 2021 and is expected to be completed by March 2022 (or six months after individual site activation).

## Supplementary Information

**Additional file 1.** Infants_PostpartumICF_Munoz et al.

**Additional file 2.** Pregnancy_and_InfantsICF_Munoz et al.

**Additional file 3.** Postpartum_WomenICF_Munoz et al.

## Data Availability

Data collected for the study will be made available to others as a de-identified patient data set after finalization of clinical study report at the discretion of the IDCRC. Analyses of data, including data from staged analyses, will be available for presentation at scientific meetings and publication to inform the scientific community. If preliminary analyses are considered of public health importance or relevant to inform research, development, and implementation of SARS-CoV-2 vaccine in pregnancy, results may be shared with public health officials and partners to inform the global scientific community. The study will be conducted in accordance with the NIH Public Access Policy publication and data sharing policies and regulations. To request study data once complete, contact Flor M. Munoz, florm@bcm.edu.

## Abbreviations

EUA: Emergency Use Authorization
U.S.: United States
ICU: Intensive Care Unit
NICU: Neonatal Intensive Care Unit
GCP: Good Clinical Practice
IRB: Institutional Review Board
OHRP: Office of Human Research Protections
FWA: Federal Wide Assurance
IDCRC: Infectious Diseases Clinical Research Consortium
COU: Clinical Operations Unit
SAE: Serious Adverse Event
AESI: Adverse Events of Special Interest
PCR: Polymerase Chain Reaction
IgG: Immunoglobulin G
IgA: Immunoglobulin A
PBMC: Peripheral Blood Mononuclear Cells
CDC: Centers for Disease Control and Prevention
FDA: Food and Drug Administration
ACOG: American College of Obstetricians and Gynecologists
DART: Development and Reproductive Toxicology
