# Supplementary material for: Multi-site observational maternal and infant COVID-19 vaccine study (MOMI-vax): a study protocol
Source: BMC Pregnancy Childbirth. 2022 May 12;22:402. doi: 10.1186/s12884-022-04500-w (PMC9096328; doi:10.1186/s12884-022-04500-w)
Supplement: Supplementary file 1 — Additional file 1. Infants_PostpartumICF_Munoz et al. [file 12884_2022_4500_MOESM1_ESM.pdf]

Institutional Review Board
Informed Consent Document for Research
MASTER CONSENT

Study Title: Observational, Prospective Cohort Study of the Immunogenicity and Safety of SARS-CoV-2 Vaccines Administered during Pregnancy or Postpartum and Evaluation of Antibody Transfer and Durability in Infants
Version Date: V2.0, May 21, 2021

Part 1 of 2: MASTER CONSENT – Infants of Postpartum Participants

Your baby is being invited to take part in a research study about the disease caused by SARS-CoV-2 virus called COVID-19. This study is a multi-site study, meaning it will take place at several different study locations. This consent form includes two parts. Part 1 is the Master Consent and includes information that applies to all study sites. Part 2 is the Study Site Information and includes information specific to the study site where you and your baby are being asked to enroll. Both parts together are the consent form and must be provided to you.

KEY INFORMATION:
Your baby is being asked to take part in a research study. Participation is voluntary. This section will give you key information to help you decide whether to participate. Detailed information can be found after this section. Ask the research team any questions you have about the study. This study is sponsored by the National Institute of Allergy and Infectious Diseases (NIAID) Division of Microbiology and Infectious Diseases (DMID).

Approximately 1,000 pregnant and postpartum women and 1,000 infants will be in this study. This observational study is designed to collect clinical information and blood samples to determine how much protection is available to the mother and their babies after receiving a COVID-19 vaccine postpartum. The study will also collect COVID-19 vaccine safety information.

Your baby is being asked to take part in this study because you recently gave birth, and you have received or are scheduled to receive, a COVID-19 vaccine. **COVID-19 vaccines are NOT given as part of this study.** You will be asked to sign a separate consent form for yourself.

Your baby will have 3 study visits. You and your baby's follow-up visits will be scheduled together whenever possible; participation in this study will last up to 12 months after delivery. You may withdraw your baby from the study at any time if you choose.

Study visits may include:
- Questions about your baby's medical history, medications and vaccinations, history of respiratory illnesses, and delivery information
- Baby's growth measurements
- Brief physical exam, if needed
- Blood samples

Study Title: Observational, Prospective Cohort Study of the Immunogenicity and Safety of SARS-CoV-2 Vaccines Administered during Pregnancy or Postpartum and Evaluation of Antibody Transfer and Durability in Infants
Version Date: V2.0, May 21, 2021

Others may benefit in the future from information learned from the study.

The possible risks of participating in this study include those associated with having samples collected and the possibility of a breach of confidentiality.

BACKGROUND:
Your baby is being asked to take part in this study because you recently gave birth, and you have received or are scheduled to receive a vaccination series of any licensed or Emergency Use Authorization (EUA) COVID-19 vaccine. **The COVID-19 vaccine is not given as part of this study.**

While COVID-19 vaccines are being administered to women during pregnancy and post-partum, there is a need to understand the effect of maternal vaccination on the infant, including the possibility that infants could be protected from COVID-19. This study will help better understand how vaccination in pregnancy and in the post-partum period could benefit mothers and their infants.

By signing this consent, you are agreeing to your baby's participation. Medical information for your infant is important to the study, so you will be asked to sign a release of medical records authorization form (this is what is on next page) for your infant to allow study staff to obtain necessary medical and delivery information.

RISKS OF THIS STUDY:
<u>Blood draws</u>: Pain, redness, or bruising at the site of the needle stick; rarely an infection. Your infant may cry while blood is collected.

<u>Breach of confidentiality</u>: The risk of breach of confidentiality related to your baby's participation in this study is very low. Precautions are taken to protect the privacy of your baby's personal information, including removing information that could be used to identify your baby. However as with any study, there is a potential risk of loss of confidentiality of your baby's information.

POTENTIAL BENEFITS:
You may receive results of the blood and breast milk tests done as part of this study by the time the study ends. Also, the knowledge gained from this study may be used to inform future policy recommendations and personal decision-making about the use of approved COVID-19 vaccines in pregnant and lactating women. There is potential benefit to society resulting from insights gained from participation in this study due to the ongoing threat of the COVID-19 outbreak.

Study Title: Observational, Prospective Cohort Study of the Immunogenicity and Safety of SARS-CoV-2 Vaccines Administered during Pregnancy or Postpartum and Evaluation of Antibody Transfer and Durability in Infants
Version Date: V2.0, May 21, 2021

Your baby does not have to be in this research study. You may choose for your baby not to be in this study without changing your healthcare, services, or other rights. Your baby can stop being in this study at any time. If we learn something new that may affect the risks or benefits of this study, you will be told so that you can decide whether or not you still want to be in this study.

STUDY PROCEDURES:
Screening/Enrollment Visit:
If you agree to be in the study, you must first sign an informed consent form for yourself and this separate consent form for your baby. We will then:

- Ask you to sign a release of medical records authorization form for your baby
- Ask you about your baby's plans for pediatric care and ask you to provide authorization for release of pediatric medical records
- Review your baby's medical records
- Ask for your baby's name, sex, and date of birth
- Ask you about your baby's medical, medication, vaccination, respiratory history including COVID-19

2, 6, and 12 Months Follow-up Visits (approximately one hour):
At these visits, we will repeat the following at each visit:

- Ask you about your baby's medical, medication, vaccination, respiratory history including COVID-19
- Measure your baby's growth
- Perform a brief physical exam, if needed
- Obtain about 1 teaspoon of blood from a vein (preferred) or by heel stick (months 2 and 6 visits)

Unscheduled visits may take place as needed (for example if a sample is missed or there is an issue with processing a sample).

REASONS FOR INVESTIGATOR WITHDRAWAL:
Your baby may be removed from the study if:

- the study would present a health risk to your baby
- your baby fails to follow the study requirements
- the study stops or
- Your baby is no longer eligible for the study.

Study Title: Observational, Prospective Cohort Study of the Immunogenicity and Safety of SARS-CoV-2 Vaccines Administered during Pregnancy or Postpartum and Evaluation of Antibody Transfer and Durability in Infants
Version Date: V2.0, May 21, 2021

PARTICIPANT WITHDRAWAL:
Your participation in this study is completely voluntary. Your baby can stop at any time. There is no penalty or loss of any benefits to which your baby is otherwise entitled if you choose not to enroll, stop, or change your mind. Always tell the study staff if you wish to stop. They will discuss any concerns about your baby's safety, and whether you need any follow up or medical care.

SOURCE OF FUNDING:
This study is being funded by the NIH, NIAID, Division of Microbiology and Infectious Diseases. The research investigator is not being paid directly by the Sponsor. A payment will be made to the study center to cover the costs of the study procedures and the services provided by the research investigator and the study staff.

LABORATORY TESTING OF BLOOD AND BREAST MILK SAMPLES:
Research tests will be done on blood samples collected from your baby to measure immune response to COVID-19 vaccines. This means that we will measure your baby's antibodies, which are proteins that the body uses to fight off the virus. We will also look at how different cells of your baby's immune system help to fight the virus. The results of these tests are useful only for research purposes. No genetic testing will be done.

Blood samples for these research tests may be sent to a central storage facility then sent to the research testing laboratories. The samples will not be labeled with your baby's name or initials, or any other information that could readily identify your baby. The samples will be labeled only with a barcode and a unique tracking number (ID code) to help protect your baby's confidentiality. Staff at the central storage facility and research testing laboratories will not know your baby's identity, or even the study identifier your baby was assigned.

The clinical study staff will keep a list in a secure area with your baby's name, contact information and the ID code (called a code key) that can link the samples to your baby, if needed. Access to the code key is limited to study staff working at the research site where your baby's blood samples were collected.

STUDY RESULTS:
Individual research results may be provided to participants by the end of the study, but they will not be made available to your baby's regular doctor and will not be placed in your baby's medical record.

Results from this study may be published while the study is ongoing and at the end of the study. Your individual identity will not be used in any reports or publications resulting from this study.

SECONDARY RESEARCH:
What happens to the blood and breast milk samples after testing?
After all tests required for this study are done, we will save your baby's leftover blood samples at a central storage facility for possible secondary research. These samples may be shared for secondary research with investigators at the site, or with researchers at other sites or other institutions. Secondary research is research that is not part of this study and is not planned yet. There is no time limit on how long these samples will be stored.

Importantly, the samples and the information we collect about your baby will not be labeled with personal identifiers; they will only have an ID code on them. Reports from secondary research will not be kept in your baby's health records or shared with you, unless required by law.

You do not have to agree to let us to save your baby's samples for secondary research. If you choose not to let us to save these samples for secondary research, they will be destroyed when the study is over. Your baby's medical care at this clinic will not be affected if you do not let us to save your baby's samples for secondary research.

If you decide at any time that you do not want the samples stored for secondary research, you must contact study staff in writing who will then notify the laboratory/storage facility. They will mark your baby's samples with a "Destroy" label and destroy them at the end of this study, or laboratory staff will take the samples out of storage and destroy them as soon as possible.

INFANT SAMPLES FOR SECONDARY RESEARCH
Please **INITIAL** whether or not **your baby's samples** may be kept for use in secondary research:

______YES, my baby's samples may be kept for secondary research.

______NO, my baby's samples may not be kept for secondary research.
